# Poster Session II - A194 COMBINING SYMPTOMS AND ENDOSCOPIC FINDINGS TO PREDICT GERD DIAGNOSED BY 24-HOUR PH MONITORING

**DOI:** 10.1093/jcag/gwaf042.194

**Published:** 2026-02-13

**Authors:** Y Han, Y Alotaibi, S Alobaid, A Alhazmi, A A Saqah, L Alrabghi, M Ismail, O Pacyna, N Anwar, R Elzaanoun, M R Jouid, E Ogunsakin, K McIntosh, R Mortuza, R Sedano

**Affiliations:** Western University Schulich School of Medicine & Dentistry, London, ON, Canada; Western University Schulich School of Medicine & Dentistry, London, ON, Canada; Western University Schulich School of Medicine & Dentistry, London, ON, Canada; Western University Schulich School of Medicine & Dentistry, London, ON, Canada; Western University Schulich School of Medicine & Dentistry, London, ON, Canada; Western University Schulich School of Medicine & Dentistry, London, ON, Canada; Western University Schulich School of Medicine & Dentistry, London, ON, Canada; Western University Schulich School of Medicine & Dentistry, London, ON, Canada; Western University Schulich School of Medicine & Dentistry, London, ON, Canada; Western University Schulich School of Medicine & Dentistry, London, ON, Canada; Western University Schulich School of Medicine & Dentistry, London, ON, Canada; Western University Schulich School of Medicine & Dentistry, London, ON, Canada; Western University Schulich School of Medicine & Dentistry, London, ON, Canada; Lawson Health Research Institute, London, ON, Canada; Western University Schulich School of Medicine & Dentistry, London, ON, Canada

## Abstract

**Background:**

Gastroesophageal reflux disease (GERD) is a common gastrointestinal disorder. Although many patients with GERD are diagnosed empirically, some require definitive testing via 24-hour pH monitoring. However, in Southwestern Ontario, there is a long wait time for pH studies. According to the Lyon Consensus, conclusive evidence for GERD includes LA grade B–D esophagitis, Barrett’s esophagus, and peptic strictures. These findings are uncommon during endoscopy and therefore apply to a limited subset of patients. Other endoscopic findings and symptoms alone are insufficient for diagnosis. Previous research has evaluated the predictive ability of these factors individually, but not in combination.

**Aims:**

We aimed to investigate the predictive value of combined clinical symptoms and endoscopic findings for GERD diagnosis confirmed by 24-hour pH monitoring.

**Methods:**

We retrospectively analyzed de-identified pH study data from patients assessed at St. Joseph’s Hospital (London, Ontario) between 2014 and 2025 using a motility database. GERD was defined as acid exposure time >6% or a DeMeester score >14.7. Associations between clinical/endoscopic variables and a positive pH test were assessed via Chi-square tests.

**Results:**

Clinical and endoscopic characteristics, along with 24-hour pH results, was analyzed for 718 patients. The following variables were significantly associated with a confirmed GERD diagnosis: heartburn (p < 0.001), regurgitation (p < 0.001), BMI ≥30 (p = 0.010), PPI responsiveness (p = 0.015), and hiatal hernia on endoscopy (p = 0.001). These five variables were combined to create a summative predictive score for each patient, ranging from 0 to 5. There was a positive correlation between the total score and the likelihood of confirmed GERD. Among patients with all five characteristics (score = 5), 80% had GERD confirmed by pH monitoring, compared to 33.14% of those with only one characteristic (p < 0.001).

**Conclusions:**

The presence of heartburn, regurgitation, BMI ≥30, PPI responsiveness, and hiatal hernia on endoscopy is strongly associated with conclusive GERD diagnosis by 24-hour pH monitoring. Patients with all of these features may be able to forgo pH studies, potentially reducing wait times for motility studies.

A194 Table 1: Proportion of positive pH tests by predictive score

None

**Table:** 

Number of concurrent variables exhibited	Negative pH test (%)	Positive pH test (%)	Total
0	118 (66.29)	60 (33.71)	178
1	114 (67.86)	54 (32.14)	168
2	87 (56.49)	67 (43.51)	154
3	64 (48.12)	69 (51.88)	133
4	28 (40.00)	42 (60.00)	70
5	3 (20%)	12 (80.00)	15
Total	414 (57.66)	304 (42.34)	718

